# Using bootstrapped quantile regression analysis for small sample research in applied linguistics: Some methodological considerations

**DOI:** 10.1371/journal.pone.0210668

**Published:** 2019-01-14

**Authors:** Larisa Nikitina, Rohayati Paidi, Fumitaka Furuoka

**Affiliations:** 1 Faculty of Languages and Linguistics, University of Malaya, Kuala Lumpur, Malaysia; 2 Faculty of Arts and Social Sciences, University of Malaya, Kuala Lumpur, Malaysia; 3 Asia–Europe Institute, University of Malaya, Kuala Lumpur, Malaysia; University of Aveiro, NEW ZEALAND

## Abstract

Quantitative applied linguistics research often takes place in restricted settings of an intact language classroom, workplace, phonetics laboratory or longitudinal sample. In such settings the samples tend to be small, which raises several methodological problems. The main aim of the current paper is to give a detailed explanation of methodological and practical implications inherent in a robust statistical method called bootstrapped quantile regression (BQR) analysis. Importantly for applied linguistics research, the BQR method could help to deal with methodological difficulties inherent in small sample studies. The current study employed a moderately small sample (*N* = 27) of students learning the Japanese language in a Malaysian public university. It examined the relationships between the students’ language learning motivation (specifically, integrative orientation), the students’ images or stereotypes about Japan and their global attitudes toward the target language country and its people. The findings indicated that there was a statistically significant relationship between the students’ attitudes toward the target language country and their integrative orientation. In addition, these attitudes were found to be the most constant determinant of the integrative orientation. Besides the applied linguistics research, the BQR method can be used in a variety of the human sciences research where a sample size is small.

## Introduction

Quantitative research in applied linguistics often takes place in restricted settings. This can be a language classroom, a workplace, a laboratory or even a longitudinal population sample. In such studies the number of observations tends to be small. Therefore, a statistical analysis cannot be based on the law of large numbers because the underlying distributional assumptions might not be met in a small sample study. If a researcher disregards the necessity to satisfy each and every of the associated assumptions the resultant study may inadvertently fill the scholarly literature with nonreplicable findings and eventuate in a replication crisis [1, 2]. In other words, repeated violations of underlying distributional assumptions in a small sample study have large scale negative consequences.

In order to obtain replicable empirical evidence applied linguistics researchers need to be aware of a number of issues associated with the choice for a statistical method. This need has been highlighted by several researchers in the field [3–7] who noted that statistical results from small sample studies might be biased and this has serious implications for the stability of findings. However, there are wider implications inherent in the cases when studies with smaller samples make inferences to larger populations. This is a major problem that concerns any statistical analysis. Therefore, it is imperative that applied linguists have a better understanding of these inherent problems so that they can select an appropriate statistical method.

A useful heuristic tool to understand issues regarding statistical inference could be the “*known—unknown*” continuum discussed in philosophy [8]. This is because this continuum can help to demonstrate linkages between a smaller sample (i.e., the *known*) and a larger population (i.e. the *unknown*) and assist in evaluating appropriateness of a statistical procedure. To be more specific, feasibility of a statistical method can be illustrated by the relationship between *known* (the sample) and *unknown* (population). Four types of statistical analyses can be identified along the “*known—unknown*” continuum. They are: (1) the analysis of “*known unknown*”, (2) the analysis of “*known known*”, (3) the analysis of “*unknown unknown”* and (4) the analysis of “*unknown known*”.

In the first “*known unknown*” type, the analysis of what we (already) know is based on what we have (yet) to know. The first law of large numbers or the weak law of large numbers allows establishing a “bridge” from the unknown parameter to the known statistic. In other words, the first law of large numbers might help to ensure that a sample statistic is found in a close vicinity to a population parameter when the sample size tends to infinity. However, a problem with the first law of large numbers is that it can create only a one-way bridge from the population parameters to the sample statistics. The law cannot be applied for statistical inference in the opposite direction [9, 10]. In the second “*known known*” type of statistical analysis, the analysis of what we (already) know is based on what is indeed known. Therefore, if researchers could collect all the necessary information about the known sample, they would use descriptive statistics to examine the characteristics of the sample. However, the problem is that descriptive statistics cannot be employed to reject or substantiate the null hypothesis in applied linguistics and other types of research [11].

In the third “*unknown unknown*” type, the analysis of what we do not (yet) know is based on what we have yet to know. If researchers collect all the necessary information about the unknown population, then descriptive statistics would be an appropriate method to describe the characteristics of the population. This type of a statistical analysis can be described as a census study. However, the census studies are rare in applied linguistics research because they are economically expensive and practically difficult to carry out [12]. Finally, in the “*unknown known*” type, the analysis of what we do not (yet) know is based on what we already know. The second law of large numbers could be used to create the “bridge” from the known statistics to the unknown parameter by combining the first law of large numbers with Bayes’ theorem [10]. Despite the existence of well-established and feasible methods to create such “bridges” a required prior probability distribution is often not available in applied quantitative research, particularly in applied linguistics. Another problem is that in applied linguistics—and generally in the human sciences research—it is often impossible to clearly define the ‘real’ population. While this causes intense debates in some disciplines [13], researchers in the field of applied linguistics, as noted by Larson-Hall [3], seem to be content to let the issue remain rather vague.

In sum, there are several notable hindrances that prevent researchers from relying on the second law of large numbers. Obviously, a classroom-based applied linguistics research cannot rely on the law of large numbers due to the small sample size. Instead, a robust statistical method known as bootstrap method could be a viable alternative. Monte Carlo simulation is another available technique. However, in the Monte Carlo method researchers face a problem of how to define the data generating process (DGP). The strength of the bootstrap method is that it generates data by resampling and replacing the actual data and does not rely on an arbitrary process of DGP that creates imaginary data.

In the “*unknown–known*” continuum, the bootstrap method can be considered as a modified version of the “*unknown known*” type of analysis. In this method, bootstrapping is employed to create empirically generated sampling distribution (EGSD). This ‘known’ EGSD is then used to construct confidence intervals (CIs) and create a ‘bridge’ to the ‘unknown’ population parameters. Without relying on (unavailable) prior probability distributions, the ‘known’ EGSD assists researchers with outlining the ‘undefined’ or ‘unknown’ population parameters.

In recent years, the American Statistical Association (ASA) recommended departing from a simplified dichotomy between ‘significant’ and ‘non-significant’ findings. Some statisticians suggest that using CIs instead of the *p*-value for statistical inference would increase validity of the results [2]. The bootstrap method offers a straightforward approach to constructing CIs in a small sample study. Another advantage of the method is that the estimations do not need to depend on the underlying distributional assumptions. There is a growing consensus among applied linguists that in studies with a small sample size the bootstrap method would offer more reliable findings and there has been an increase in studies that employ this method [4–7].

However, it should be noted that the bootstrap method is not a panacea to solve all problems associated with a small sample applied linguistics research. This is because the method has a tendency to proliferate outliers in the estimation, which is another obstacle for producing valid empirical results [6, 14]. In order to overcome this problem, applied linguistic researchers might want to combine bootstrapping with a novel for the discipline method called Quantile Regression (QR). The QR analysis, which is based on a quantile value and more robust against the presence of extreme values [15], was introduced in educational research by Chen and Chalhoub-Deville [16].

To sum up the argument thus far, a small sample applied linguistics research could suffer from two major methodological problems: the violation of underlying distributional assumptions and the presence of outliers in the dataset. The bootstrapped quantile regression (BQR) could be an appropriate statistical approach to mitigate these problems because this robust method combines the methodological advantages of bootstrapping and the merits of quantile regression. This method was only recently introduced in applied linguistics research by Nikitina and Furuoka [6]; however, due to a limited space allocated to the original “forum piece” publication, the statistical reasoning behind the method and a detailed explanation of the actual procedure had to be left out. Therefore, the main motivation behind the current study is to provide statistical and methodological reasonings behind the BQR method and give a detailed explanation about why and how this method can be employed in applied linguistics studies with a small sample. As in our previous study [6], the focus is on psychological variables—attitudes and language learning motivation (L2 motivation)—involved in the process of learning an additional language. The present study used new data to examine the relationship between language attitudes and L2 motivation (specifically, integrative orientation) in a moderately small sample of 27 (*N* = 27) learners of the Japanese language in a Malaysian public university.

### Origins of the integrative orientation concept and problems with its measurement

Pioneering research by Canadian psychologists Robert C. Gardner and Wallace E. Lambert [17, 18] has highlighted an important role that motivation plays in language learning. The researchers introduced the concept of ‘integrative orientation’ within the L2 motivation. Earlier studies by Gardner and Lambert culminated in the influential socio-educational theory of L2 motivation [19] where the integrative orientation evolved from the originally proposed notion of language learners’ feelings of identification and affiliation with the target language community [17, 18] to a wider set of language learners’ attitudes and dispositions toward the target language itself and the communities and countries where this language is spoken.

Despite several decades of intensive research on L2 motivation, the integrative orientation remains one of the most elusive concepts and is notoriously difficult to measure [20, 21]. This can be partially due to a complex nature of human psychological processes. It also can be due to the methodological choices made by researchers. Psychometric surveys on L2 motivation usually consist of close-ended statements with attached Likert-type scales [17, 18]. These statements are preselected by researchers and leave no space for the participants to express their own attitudes and motivational drives. It should be noted that applied linguistics research on language attitudes rarely employs the full range of approaches developed in psychology, such as the thermometer-type scales or the mental images and stereotypes [22] to collect the data. The current study addresses this methodological oversight. Moreover, it uses a novel recently introduced robust statistical procedure [6] to analyse the data.

### Robust statistics in applied linguistics research

In recent years, there has been an upward trend in applied linguistics research that employs robust statistical methods. Among the earlier studies, Larson-Hall and Herrington [4] convincingly demonstrated that robust statistical tests could be more powerful and accurate than parametric tests when data are not normally distributed and sample size is small. Using real datasets and employing both parametric and non-parametric statistics the researchers compared differences in pronouncing /*r*/ and /*l*/ between three groups of Japanese learners of English and speakers of English as the first language (L1 speakers). A parametric one-way ANOVA test was able to detect a significant difference in the pronunciation between only one group of English language learners and speakers of English. In contrast, robust statistics (bootstrapping) detected a statistically significant difference in the pronunciation between all the three groups involved in the study [4].

In another article, Larson-Hall [5] compared the findings from the ‘classical’ statistical tests, such as the *t*-test, one-way ANOVA and correlation analysis, with the results gained from the robust versions of these tests. She found considerable discrepancies in some of the results. Thus, the *p*-value obtained in the classical *t*-test was .067 (indicating a lack of statistically significant difference) while the *p*-value from the robust *t*-test was .030 (which pointed to a statistical significance). Similarly, the correlation coefficient *r* in the classical correlation test was .07 (and rather small) while the coefficient in the robust correlation procedure was .22 (and indicated a stronger association between the variables). Only in the case of the one-way ANOVA test and its robust variant the researcher could find no differences in the findings.

A robust statistical procedure known as bootstrapping has been employed in applied linguistics studies with a small sample size as a means to deal with non-normality of the data sets and in order to construct confidence intervals (CIs). For example, Gallagher [23] examined relationships between cross-cultural adaptation and willingness to communicate in the second language among Chinese speaking international students in a British university. To address the problem of non-normal data, the researcher performed the SEM analysis using the bootstrap method. This helped to enhance goodness-of-fit estimators in the SEM analysis. The findings of the study [23] indicated a statistically significant linkage between the variables of interest. Hessel [24] applied the bootstrapping procedure to compute CIs in the regression analysis of the relationships between an ideal second language (L2) self as projected by German learners of English and efforts that these learners expended toward the attainment of the ideal L2 self. The researcher detected a statistically significant relationship between the two variables.

In a similar way, Gu and Cheung [25] applied the bootstrap method to estimate CIs in their study of the relationships among ethnic minorities schoolchildren’s perceptions of an ideal L2 self, the children’s acculturation into the host society, the role of the heritage culture and the efforts that the children expended to learn the mainstream language. As the researchers noted [25], the bootstrap method was appropriate for the mediation analysis because it does not impose distribution assumptions. Zhang [26] used bootstrapping in the SEM analysis of the relationships between young Chinese-speaking language learners’ morphological awareness and their reading comprehension. The researcher found that the learners’ morphological awareness had a statistically significant direct effect on their reading comprehension.

Quantile Regression (QR) is another robust statistical method that could be fruitfully employed in applied linguistics research. The method was first introduced in general educational research by Chen and Chalhoub-Deville [16] who examined the relationship between young children’s language reading ability and their performance in mathematics. However, despite its methodological advantages the method was not adopted by applied linguistics researchers until recently. For example, Nikitina and Furuoka [6] included QR as one of the methods to analyse the relationships between Russian language learners’ attitudes toward the target language country and its people and the students’ stereotypes about Russia. The researchers first performed the OLS analysis which was followed by the bootstrapping and the QR methods before introducing the bootstrapped quantile regression (BQR). As their findings indicated, the OLS and QR methods failed to detect statistically significant relationships between the variables. This result was counter-intuitive and did not support major and influential psychological theories. However, the findings from the BQR method were able to detect a statistically significant relationships between the language learners’ attitudes toward Russia and their stereotypes about the target language country [6]. The discrepancies in the results highlight the importance of Larson-Hall’s observation that the differences in the findings obtained from different statistical analyses of the same data indicate the need for enlightened and well-reasoned statistical approaches [5].

### Statistical reasoning behind the BQR method

From a statistical methodological perspective, a majority of applied linguistics studies employ parametric statistical methods. Bivariate ordinary least squares (OLS) analysis, which is among the most widely employed methods in the human sciences, is based on the following equation [27, 28]:

$$y_{i}=\beta_{0}+\beta_{1}x_{i}+\varepsilon_{i}$$ (1)

where *y*_*i*_ is the dependent variable, *x*_*i*_ is the independent variable, *β*_*0*_ is the intercept, *β*_*1*_ is the slope coefficient and *ε*_*i*_ is the error term. For example, if *y*_*i*_ is a foreign language learner’s academic performance and *x*_*i*_ is his or her motivation to learn this particular language, a statistical significance of the slope coefficient (*β*_*1*_) indicates that the language learner’s motivation had a statistically significant impact on the academic performance. In general terms, a multivariate OLS model in a matrix form can be expressed as [27, 28]:

y=Xβ+ε (2)

where *y* is the 1 × *n* vector of the dependent variable, *X* is the *n* × *K* matrix of the independent variable, *β* is the *K* × 1 vector of the slope coefficients, *ε* is the 1 × *n* vector of the error terms, *n* is the number of observations and *K* is the number of independent variables. For example, suppose that *y* is a foreign language learning motivation (L2 motivation) and *X* are the factors (e.g., professional or travel needs) which are assumed to have some impact on L2 motivation. A statistical significance of the slope coefficient (*β*) would indicate that these factors had a statistically significant impact on L2 motivation. In other words, the slope coefficient is empirically tested to determine whether it is significantly different from zero. Formally, the null hypothesis and the alternative hypothesis for the OLS estimate of the *k*th slope coefficient (*b*_*k*_) can be expressed as [27, 28]:

$$\begin{array}{l} H_{0}:b_{k}={\bar{\beta }}_{k}\\ H_{1}:b_{k}\neq {\bar{\beta }}_{k} \end{array}$$ (3)

where ${\bar{\beta }}_{k}$ is the unknown and hypothesised value of the *k*th slope coefficient which is specified by the null hypothesis. The null hypothesis typically specifies that the estimated coefficient is equal to zero. Under the null hypothesis of $H_{0}:b_{k}={\bar{\beta }}_{k}$, the *t*-ratio can be used for the hypothesis testing and calculated as [27, 28]:

$$t_{k}=\frac{b_{k}-{\bar{\beta }}_{k}}{SE\left(b_{k}\right)}=\frac{b_{k}-{\bar{\beta }}_{k}}{\sqrt{s^{2}({\left(X^{\prime }X\right)}^{-1}}{)}_{kk}}=\frac{b_{k}-{\bar{\beta }}_{k}}{\sqrt{s^{2}SS^{kk}}}$$ (4)

where SE(*b*_*k*_) is the standard error of the OLS estimate of the *k*th slope coefficient, *s*^*2*^ is the OLS estimate of residual variance, ((*X*′*X*)^−1^)_*kk*_ is the *k*th diagonal element of the inverse matrix of *X*′*X* which also can be expressed as *SS*^*kk*^. Eq (4) clearly establishes that the distribution of the *t*-ratio hinges on a close relationship between the population parameter *β* and sample statistic *b*. In a large sample study, the Central Limit Theorem (CLT) would play a central role in establishing a ‘bridge’ between the two. In other words, under the Lindeberg–Lévy CTL theorem, the probability distribution of a sample statistic will eventually converge in a desirable distribution [27, 28]. More formally, if the random variables *x*_*t*_ are independently and identically distributed (IID), the random variable *z* will *converge in distribution* to a normal distribution when the sample size tends to infinity [27, 28]:

$$z\equiv \frac{1}{\sqrt{n}}\Sigma_{i=1}^{n}x_{i}\overset{d}{\to }N\left(\mu ,\sigma \right)$$ (5)

where *n* is the sample size, *μ* is the population mean and *σ* is the population variance. In the context of the OLS analysis, the OLS estimator, *b*, will *converge in distribution* to a normal distribution when the sample size tends to infinity [28, 29]:

$$b\overset{d}{\to }N\left[\beta ,\sigma^{2}{\;(X^{\prime }X)}^{-1}\right]$$ (6)

where *β* is the population slope parameter and *σ*^2^(*X*′*X*)^−1^ is the population covariance matrix.

One of the main problems associated with the null hypothesis significance testing (NHST) is the dichotomy between ‘significance’ and ‘non-significance’ [3]. In order to overcome this problem, the current paper reports confidence intervals (CIs) rather than *p*-values. Under the assumption of the error term normal distribution, the CIs for the unknown population parameter (*β*) could be derived from the distribution of the known sample statistic (*b*). The formula to construct the CIs for the unknown population parameter is [28]:

$$\left[b_{k}-t_{\alpha /2}\sqrt{s^{2}SS^{kk}},b_{k}+t_{\alpha /2}\sqrt{s^{2}SS^{kk}}\right]$$ (7)

where *α* is the significance level. The null hypothesis cannot be rejected if the hypothesised value (${\bar{\beta }}_{k}$) is included in the confidence interval.

As mentioned earlier in this article, analytical methods that assume a large sample are not usually feasible for applied linguistics research where the sample size is small. In other words, in small sample studies statistical analyses cannot be based on the Central Limit Theorem (CLT) because the theorem applies exclusively to large samples. Without this theoretical support, a small sample distribution of the OLS estimator *b* is, essentially, unknown [28–30]. A basic premise in the OLS analysis, such as the Gauss–Markov theorem, does not permit drawing inferences about the unknown population parameter *β* in the population regression function (PRF) from the sample statistic *b* in the sample regression function (SRF) [30]. In the context of the ‘*known–unknown*’ continuum, the main problem of the small sample research is that there is a missing ‘bridge’ that links the ‘known’ sample statistic to the ‘unknown’ population parameter.

This means that in a small sample research there will always remain an unfilled ‘gap’ between the unknown population parameter and the unknown sample statistic. This gap can be reduced by imposing the normality assumption of error terms in the PRF, which will permit obtaining a small sample distribution of the OLS estimator. In addition, applied linguistics researchers need to examine the appropriateness of all imposed assumptions for a small sample research [29, 30]. This implies the necessity to empirically establish whether the error terms are normally distributed with zero mean, constant variance and no correlation [28–30]. However, the problem arises when a researcher cannot empirically establish the normality assumption. In such cases the question arises: How this methodological problem can be remedied if or when the normality assumption of error terms cannot be empirically established? To remedy methodological problems inherent in a small sample study, Nikitina and Furuoka [6] suggested to combine two robust statistical methods, namely, the bootstrap method and the quantile regression (QR) analysis [6]. The bootstrap method is a robust statistical procedure which could be employed for a small sample analysis without relying on the error terms normality assumption [31]. In this method, the standard deviation of the slope coefficients is created by the bootstrap re-sampling method [32, 33]. The bootstrapped standard error is estimated following these three steps [34–37]:

1. draw a large number (*R*) from the bootstrapped samples (y*) by a resample method with replacement, say y*(1)…….y*(R).
2. estimate the bootstrapped slope coefficient (b*) from each of the bootstrapped samples, say b*(1)…….b*(R).
3. calculate the standard error (*SE*) from the distribution of the bootstrapped slope coefficients.

The bootstrapped standard error can be calculated as [34–38]:

$$SE\left(b^{*}\right)={\left(\frac{\sum_{k=1}^{R}{\left(b_{k}^{*}-b^{*}\left(m\right)\right)}^{2}}{R-1}\right)}^{1/2},\;b^{*}\left(m\right)=\frac{\sum_{k=1}^{R}b_{k}^{*}}{R}$$ (8)

where b*(m) is the mean value of the bootstrapped slope coefficient in all the bootstrapped samples, b* is the bootstrapped OLS estimate of the slope coefficient, *SE* is the standard error, *R* is the number of repetitions, $b_{k}^{*}$ is the bootstrapped estimate of the slope coefficient from the bootstrapped sample y*(k).

The QR is another efficacious robust statistical method. However, while the bootstrapping has become an established method in applied linguistics research [7], QR has been largely overlooked by the researchers. Unlike the OLS estimation, where the relationships between independent (*x*) and dependent variables (*y*) are based on the conditional mean function, *E(y|x)*, and where the OLS minimizes the sum of squared errors (SSE), the QR analysis is based on the conditional quantile function, *Qq(y|x)* [33, 34]. The QR estimate of the slope coefficient will minimise the following objective function [39]:

$$Q_{q}\left(\beta \right)=\Sigma_{i=1}^{N}\left|y_{i}-\beta x\right|$$ (9)

where *β* is the slope coefficient, *q* is the quantile value and *N* is the number of observations. As Eq (9) shows, the QR minimises the sum of absolute errors (SAE) and, therefore, this method is more robust against outliers compared to the OLS [35]. In sum, in the BQR method, the methodological advantages of the QR procedure are amplified by the bootstrapping [6].

Since the OLS analysis remains one of the most widely used methods in applied linguistics research, the current study included this analysis with the aim to demonstrate the main arguments and methodological reasonings put forward here. It should be noted that in order to allow for the application of the OLS analysis, researchers need to perform diagnostic tests that establish whether the error terms are normally distributed, have constant variance and no correlation [28–30]. In the current study, the following three main diagnostic tests assessed the distribution of error terms: the normality test, the heteroscedasticity test (to establish whether variance is constant) and the autocorrelation test (to assess whether the error terms are correlated).

The assumptions of zero mean, constant variance and no correlation are crucial for establishing the legitimacy of the method, hypothesis testing and statistical inference of the findings from a smaller sample to a larger population. As Larson-Hall [3] noted, all parametric tests are based on a number of assumptions and the data need to meet these assumptions to ensure that the selected test is appropriate. In other words, a violation of *any* of the underlying assumptions jeopardizes the hypothesis testing based on confidence intervals or *p*-values. In the current study, three diagnostic tests were performed to assess the assumptions of the OLS analysis. Firstly, the Jarque–Bera test assessed whether the error terms were normally distributed [40]. The null hypothesis in this test is that the error terms in the population regression function (PRF) are normally distributed. If the test rejects the null hypothesis, the error terms have non-normal distribution. Secondly, the Breusch–Pagan–Godfrey test established whether the error terms had constant variance [41, 42]. The null hypothesis in this test is that the error terms have constant variance. If the test rejects the null hypothesis, the error terms do not have constant variance. Thirdly, the Breusch–Godfrey test [43, 44] assessed whether the error terms were correlated. The null hypothesis in this test is that the error terms in the PRF are not correlated. If the test rejects the null hypothesis, the error terms are correlated. The diagnostic tests for the OLS analysis were conducted using the EViews statistical package. Furthermore, researchers must consider a possibility that there are outliers in the dataset and that these outliers will affect the findings. Outliers are defined as cases that are considerably different from the rest of the data [3]. In the current study, the hat matrix test was conducted to detect outliers. A methodological advantage of this test is that it gives a visual display of the findings [6]. The hat matrix test for the OLS analysis was performed with the aid of the EViews statistical package.

## Research methodology

### Participants and procedure

This project was approved by the Center for Research Grant Management, University of Malaya. The data were obtained from 27 (*N* = 27) learners of the Japanese language in a major Malaysian public university. This sample can be considered as moderately small. The participants were between 20 and 24 years old (*M* = 21.7; *SD* = 1.1). There were more female (71%) than male students in the sample, which reflects the gender ratio of students in Malaysian institutions of higher learning. Participation in this study was voluntary. The students were informed about the purposes of this study and told that returning the questionnaire forms with the answers to the researcher implied their consent to participate in this survey. Among the total 30 students in the class, 27 returned the questionnaire forms with all of the questions answered.

The questionnaire contained one open-ended question, two thermometer-type scales and five close-ended statements. The open-ended question was “What images come to your mind when you hear the words ‘Japan’ and ‘Japanese’?”. This item solicited the students’ personal or endogenous stereotypes about the target language country, Japan. In addition, the respondents were asked to mark each image on their lists from -2 (a ‘very negative’ image) to +2 (a ‘very positive’ image). This allowed gathering the students’ endogenous images of Japan, its culture, people and assessing the attitudes embedded in these images.

### Variables and measurement

The current study examined the relationship between the language learners’ integrative orientation (the variable *Integrative orientation*) and its three possible determinants, namely, the learners’ attitudes toward the target language country (the variable *Country*), their attitudes toward the target language speakers (the variable *People*) and the attitudes embedded in the personally held stereotypes about the target language country (the variable *Stereotypes*).

The variable *Integrative orientation* was measured by the following five close-ended statements (Cronbach’s alpha 0.696): “Learning the Japanese language will enable me to better understand the ways of life in Japan”; “Learning this language will enable me to appreciate Japanese art and literature”; “Learning Japanese will enable me to understand modern Japan”; “Learning Japanese will allow me to get to know its speakers better” and “I decided to learn this language because I am interested in Japanese popular culture”.

The variables *Country* and *People* were measured, respectively, by two thermometer-type scales. Such scales are widely-employed in psychology research because they allow assessing general or global attitudes held by people about various phenomena [22]. In the current study, the end points of the scales were graded as 0 °C (to mark an ‘extremely unfavourable’ attitude) and 100 °C (to mark an ‘extremely favourable’ attitude). In addition, 5 °C intervals were set between the two extreme points. The variable *Stereotypes* was assessed by the valence ratings that the students had attached to their images of Japan; these ratings ranged from -2 to +2.

### Estimation procedure

Four statistical procedures were performed, namely, the ordinary least squares (OLS) analysis, the bootstrapped OLS (BOLS) analysis, the quantile regression (QR) analysis and the bootstrapped QR (BQR) analysis. The null hypothesis of no relationship between the variables was to be rejected if zero was not included in the confidence intervals (CIs). Most of the analyses were conducted with the aid of the *R*-programming language which has emerged as a popular statistical tool in applied linguistics research [45]. Particularly, three statistical packages—the “lm” package, the “boot” package and the “quantreg” package—were essential. The “lm” package aided with the OLS analysis; the “boot” package enabled the bootstrapping procedure which constructed confidence intervals in the OLS analysis; the “quantreg” package was used for the quantile regression (QR) analysis; the bootstrapped quantile regression (BQR) analysis was conducted by the “boot” package and the “quantreg” package. The diagnostic tests and the hat matrix test in the OLS analysis were performed using the EViews statistical package. It should be noted that the findings for the estimated standard errors and confidence intervals may differ each time when using the R programming for the bootstrap analysis. However, these differences are fairly minor. A possible way to cope with this problem is to increase the number of replications in the bootstrap analysis. Using commercial software, such as the EViews statistical package, produces more consistent results for the bootstrap method when estimating the standard errors and confidence intervals.

The current study considers the 10 percent level of statistical significance; it also reports the findings at the 1 and the 5 percent levels. There is a trade-off relationship between Type I and Type II errors: an increase in the significance level from 5 percent to 10 percent would decrease a possibility of committing Type II error by approximately 20 percent [3]. Researchers and methodologists recommend that the statistical significance level in quantitative research in the Social Sciences, including applied linguistics, be set to 10 percent in order to avoid Type II error [3, 6, 46–48]. As Jenifer Larson-Hall succinctly put it in her influential book on statistical methods in second language research (p.102), “Quote me, and quote also Kline (2004) and Murphy and Myors (2004), who have argued that the alpha level should be set to *α* = .10 in the social science” [3]. The current study acknowledges these methodological considerations.

### Steps to run the R programming

The data and R programming codes are available in the “Supporting information” section of this article. To be more specific, the file S1 Table contains the data collected from the participants in this study. Each of the files S1–S4 Codes contains the R programming code to aid with statistical analysis of the data, including the OLS analysis, the BOLS analysis, the QR and the BQR procedures. These files are also available at: https://sites.google.com/site/fumitakafuruokawebpage2/home/paper-7.

Steps to run the R programming using these files are as follows: 1) download all files from the “Supporting information” section or from the website above, 2) create a folder at C:/R, 3) rename the original file “S1 Table. Data used for the analysis” to “japan” and save it as the folder C:/R/japan.txt, 4) open the R program, 5) type “library(Rcmdr)” and press the “Enter” key; 6) to conduct the OLS analysis, select all codes in the file “S1 Code. R code for OLS analysis”, copy and paste the codes in the “R Script” window, select all (Ctrl+A) and press “submit”, 7) for the BOLS analysis, select all codes in the file “S2 Code. R code for Bootstrapped OLS”, copy and paste them into the “R Script” window, select all (Ctrl+A) and press “submit”, 8) for the QR analysis, select all codes in the file “S3 Code. R code for QR”, copy and paste them into the “R Script” window, select all (Ctrl+A) and press “submit”, 9) for the BQR analysis, select all codes in the file “S4 Code. R code for Bootstrapped QR”, copy and paste them into the “R Script” window, select all (Ctrl+A) and press “submit”.

## Empirical findings

Table 1 shows findings from the OLS test. As can be seen in the table, zero was included in the CIs at all three levels of significance for the variable “*People*”. This means that the null hypothesis of no statistically significant relationship between this and the dependent variable “*Integrative orientation*” could not be rejected.

**Table 1:** Results of OLS analysis (Dependent variable: *Integrative orientation*).

Variables	Coefficient	Standard Error	Confidence Intervals (CIs)
**Country**	0.020***	0.006	*90%* [0.009, 0.030] *95%* [0.008, 0.032] *99%* [0.004, 0.036]
**People**	-0.002	0.004	*90%* [-0.009, 0.005] *95%* [-0.011, 0.006] *99%* [-0.013, 0.009]
**Stereotypes**	0.306**	0.156	*90%* [0.049, 0.563] *95%* [0.001, 0.612] *99%* [-0.095, 0.708]
**Intercept**	2.331***	0.469	*90%* [1.559, 3.102] *95%* [1.411, 3.250] *99%* [1.122, 3.539]

*** indicates statistical significance at the one percent level;

** indicates statistical significance at the five percent level

At the same time, it was found that zero was outside of the CIs at all three levels of significance for the variable “*Country*”. This means that the null hypothesis was rejected at the one percent level of statistical significance. Also, zero was outside of the CIs at first two levels of significance for the variable “*Stereotypes*”. Therefore, for this variable, the null hypothesis was rejected at the five percent level of significance.

Next, the ‘omitted’ diagnostic tests that should have been performed prior to the OLS analysis were carried out. Table 2 reports the findings.

**Table 2:** Findings from diagnostic tests.

Assumptions	Name of tests	Statistics
**Normal distribution of error terms**	Jarque–Bera test	1.076
**Constant variance in error terms (homoscedasticity)**	Breusch–Pagan–Godfrey test	0.290
**No correlation among error terms** **(no autocorrelation)**	Breusch–Godfrey test	4.609**

** indicates statistical significance at the five percent level

Firstly, the Jarque–Bera test could not reject the null hypothesis of normally distributed error terms. Therefore, the first assumption of the OLS analysis was met. Secondly, the Breusch–Pagan–Godfrey test was not able to reject the null hypothesis of homoscedasticity (i.e., constant variance of error terms); hence, the second assumption was met. Finally, the Breusch–Godfrey test rejected the null hypothesis of no autocorrelation among the error terms, which indicated that there were correlated relationships among the error terms. This means that the third assumption for the OLS procedure was violated.

**Fig 1:**
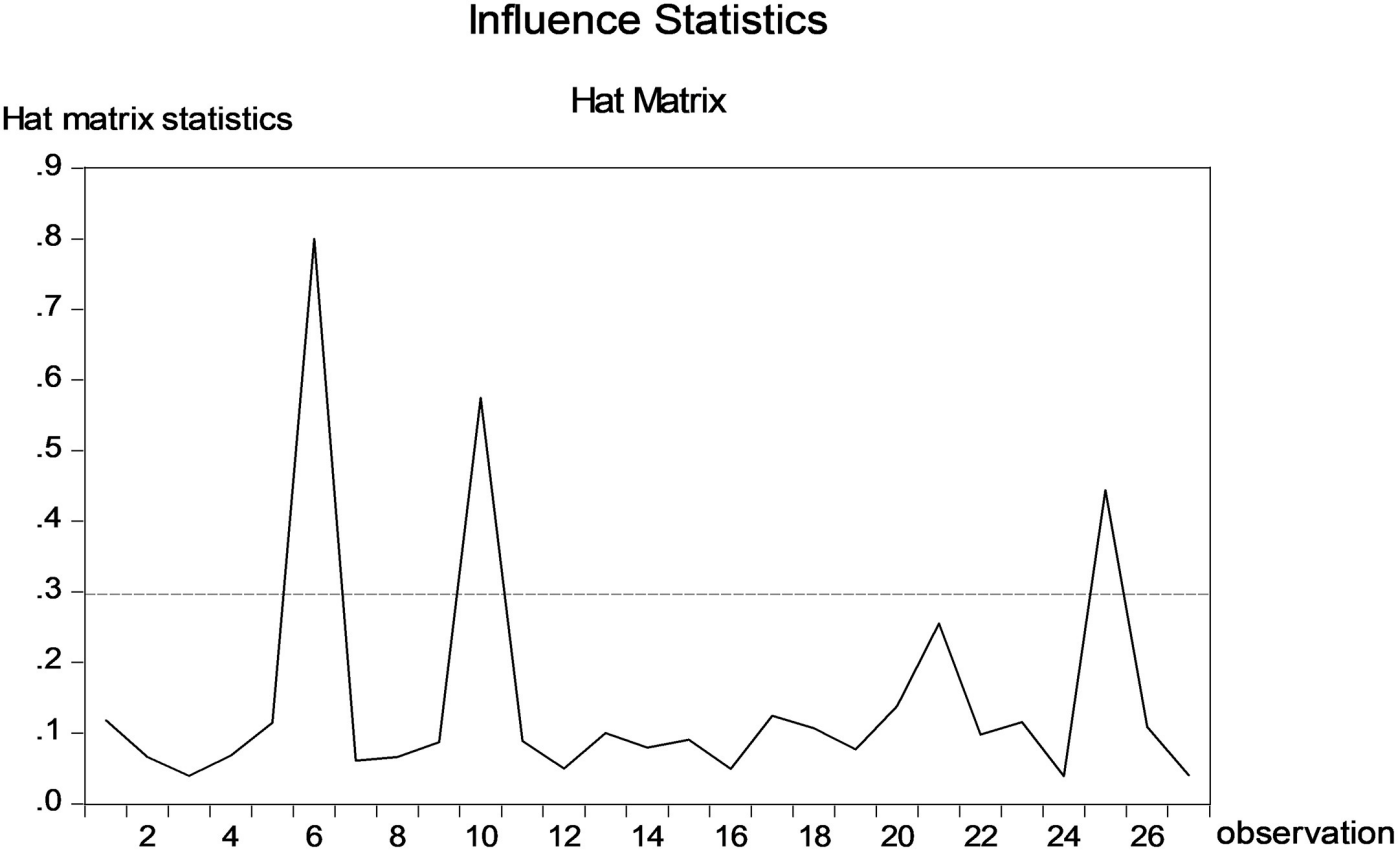
Results of the hat matrix test. *Note*: y-axis indicates the hat matrix statistics; x-axis indicates the observations.

In the next step, the hat matrix test examined whether there were outliers in the dataset. If an outlier is near the mean value of the independent variable, it would have little impact on the OLS estimation; in contrast, if an outlier is at a great distance, its effect would be substantial [49]. The hat matrix test identified three prominent outliers, namely, Observations #6, #10 and #25 (see Fig 1). Removing outliers is undesirable for three reasons. Firstly, such decisions tend to be arbitrary; secondly, if one outlier is removed other outlying cases can emerge; thirdly, removing outliers may violate assumptions of data distribution [3, 6, 50]. Therefore, the current study retained all outliers in the empirical analysis.

Since not all of the three assumptions of the OLS analysis could be met, adopting this method was not appropriate. Therefore, the study proceeded to employ robust statistical methods. The bootstrapped OLS (BOLS) analysis was carried out in the next step. The bootstrapping procedure estimated standard errors and constructed the confidence intervals (CIs). Table 3 shows the findings. As can be seen from the table, zero was included in the CIs at all three levels of significance for the variables “*People*” and “*Stereotypes*”. Therefore, the null hypothesis could not be rejected for these two variables. On the other hand, zero was outside of the CIs at first two levels of significance for the variable “*Country*”. Based on these findings, the null hypothesis for the variable “*Country*” was rejected at the five percent level of statistical significance.

**Table 3:** Findings from BOLS analysis (Dependent variable: *Integrative orientation*).

Variables	Coefficient	Standard Error	Confidence Intervals (CIs)
**Country**	0.020**	0.007	*90%* [0.007, 0.029] *95%* [0.001, 0.032] *99%* [-0.014, 0.036]
**People**	-0.002	0.009	*90%* [-0.007, 0.020] *95%* [-0.011, 0.024] *99%* [-0.025, 0.034]
**Stereotypes**	0.306	0.236	*90%* [-0.006, 0.774] *95%* [-0.078, 0.843] *99%* [-0.174, 1.026]
**Intercept**	2.331***	0.526	*90%* [1.436, 3.117] *95%* [1.274, 3.285] *99%* [0.975, 3.793]

*** indicates statistical significance at the one percent level;

** indicates statistical significance at the five percent level

Next, this study proceeded to perform the QR analysis in order to address the problem of outliers in the dataset. The findings from the QR analysis uniformly confirmed the results of the OLS method. As Table 4 shows, the QR analysis rejected the null hypothesis of no statistical significance for the variables “*Country*” and “*Stereotypes*”. At the same time, it was not able to reject the null hypothesis for the variable *“People*”. This indicates that the respondents’ attitudes toward the target language country as well as their stereotypes about Japan had a statistically significant relationship with the integrative orientation.

**Table 4:** Findings from QR analysis (Dependent variable: *Integrative orientation*).

Variables	Coefficient	Standard Error	Confidence Intervals (CIs)
**Country**	0.018**	0.008	*90%* [0.004, 0.031] *95%* [0.002, 0.034] *99%* [-0.002, 0.038]
**People**	-0.004	0.005	*90%* [-0.014, 0.005] *95%* [-0.016, 0.007] *99%* [-0.019, 0.011]
**Stereotypes**	0.536***	0.203	*90%* [0.201, 0.870] *95%* [0.17, 0.935] *99%* [0.011, 1.060]
**Intercept**	2.439***	0.611	*90%* [1.432, 3.445] *95%* [1.240, 3.638] *99%* [0.863, 4.015]

*** indicates significance at the one percent level;

** indicates significance at the five percent level

In the following step the BQR analysis was performed. Table 5 shows the findings. It is interesting to note that the findings from this analysis confirmed the results of the BOLS method. The BQR procedure rejected the null hypothesis of no statistically significant relationship between the variables “*Country*” and “*Integrative orientation*” at the 10 percent level of significance. Hence, the relationship between these variables could be considered as statistically significant. These results suggest that the respondents’ holistic attitudes toward Japan as a country had a statistically significant relationship with their integrative orientation. At the same time, the BQR test could not reject the null hypothesis for the independent variables *“People*” and *“Stereotypes*”.

**Table 5:** Findings from BQR analysis (Dependent variable: *Integrative orientation*).

Variables	Coefficient	Standard Error	Confidence Intervals (CIs)
**Country**	0.018*	0.010	*90%* [0.002, 0.032] *95%* [-0.004, 0.038] *99%* [-0.040, 0.050]
**People**	-0.004	0.014	*90%* [-0.021, 0.031] *95%* [-0.030, 0.040] *99%* [-0.040, 0.057]
**Stereotypes**	0.538	0.389	*90%* [-0.208, 1.053] *95%* [-0.288, 1.149] *99%* [-0.514, 1.290]
**Intercept**	2.439***	0.833	*90%* [1.122, 3.600] *95%* [1.000, 3.855] *99%* [0.739, 4.609]

*** indicates significance at the one percent level;

* indicates significance at the ten percent level

There were some discrepancies in the results. These discrepancies should be discussed and addressed. To be more specific, a comparison of the findings reported in Tables 1 and 3 reveals that the standard error for the variable *Country* increased from 0.006 in the OLS analysis to 0.007 in the BOLS analysis. Similarly, the standard error for variable *People* increased from 0.004 in the OLS analysis to 0.009 in the BOLS; for the variable *Stereotypes* it increased from 0.156 to 0.236. These discrepancies occurred because the presence of autocorrelation in the OLS analysis resulted in the underestimation of the standard errors. Furthermore, as Tables 1 and 4 show, though the slope coefficients for the variables *Country* and *People* had approximately same values the slope coefficient for the variable *Stereotypes* drastically increased from 0.306 in the OLS analysis to 0.536 in the QR procedure. This happened because the presence of outliers in the OLS analysis led to the underestimation of the slope coefficient. (Note: The authors are grateful to the reviewer who pointed out the increase in the slope coefficient for the variable “Stereotypes” in the QR analysis).

Also, there were also discrepancies in the patterns of significance among the different methods. For example, there were two statistically significant variables—*“Country”* and *“Stereotypes”*–in the OLS analysis. However, in the BOLS analysis only the variable *Country”* was found to be statistically significant. The QR analysis detected two statistically significant variables, namely, *“Country”* and *“Stereotypes”*, while only the variable *“Country”* was statistically significant in the BQR analysis. In view that the diagnostic tests detected a violation of the OLS assumption and the hat matrix test detected the presence of outliers, the results from the BQR analysis can be considered as the most accurate. This means that *“Country”* was the only independent variable that had a statistically significant relationship with the dependent variable “*Integrative motivation”*.

Another way to reach a definitive conclusion when there are discrepancies in the patterns of significance is conducting the effect size analysis. (Note: The authors are grateful to the reviewer who reminded us about the importance of the effect size analysis.) Effect size is a measurement of the magnitude of the impact of independent variables on a dependent variable [3]. In this study, the Cohen’s *f*^*2*^ method was used to measure the effect size [51]. In other words, this analysis enables assessing a relative magnitude and importance of the impact among the independent variables. Table 6 reports the findings from the effect size analysis. As can be seen from the table, the findings were consistent among all of the analyses; they indicated that the independent variable “*Country”* had the largest effect on the dependent variable “*Integrative Orientation*”.

**Table 6:** Effect size analysis (Dependent variable: *Integrative orientation*).

Independent Variables	Methods	
	OLS and BOLS	QR and BQR
**Country**	0.461	0.279
**People**	0.009	0.040
**Stereotypes**	0.167	0.174

Cohen’s *f*2 was used to measure the effect size. A bootstrapped method was used to estimate the standard error. Thus, the effect sizes in the OLS and BOLS methods are the same. Similarly, the effect sizes of the QR and BQR methods are the same.

It should be noted that the choice of the analytical procedure might have some impact on the strength of the effect. For example, the effect size of the variable “*Country*” was 0.461 in the OLS and the BOLS methods; it decreased to 0.279 in the QR and BQR methods. By contrast, the effect size of the variable “*People*” increased from 0.009 in the OLS and BOLS methods to 0.040 in the QR and BQR methods. Similarly, the effect size of the variable “*Stereotypes*” increased from 0.167 in the OLS and BOLS methods to 0.174 in the QR and BQR analyses.

The presence of autocorrelation in the OLS analysis might affect the effect size calculations. In this study, the bootstrap analysis has produced a new value for the standard error, however, it did not yield a new effect size. Therefore, it might be difficult for researchers to gauge the impact of autocorrelation on the effect size when using different analytical methods. Importantly, the presence of outliers also might affect the effect size calculations. For example, in the current study the effect size of the variable “*Stereotypes*” increased from 0.167 in the OLS and BOLS methods to 0.174 in the QR and the BQR methods. This fact highlights the importance of establishing whether there are outliers in the dataset.

Overall, the findings of this study highlight the prominence of language learners’ holistic or global attitudes toward the target language country. They indicate that such attitudes could be among the important determinants of the integrative orientation within L2 motivation.

## Discussion

This study has offered a detailed explanation of methodological and practical implications inherent in a novel robust statistical method called bootstrapped quantile regression (BQR) analysis. It has demonstrated how applied linguistics and more generally the human sciences research with a small sample could overcome two main methodological problems inherent in the popular OLS method, namely, the violation of the test assumptions and the presence of outliers in the dataset. These are two main contributions of the current paper. Throughout the article we highlighted the importance of observing each and every of the assumptions for statistical tests and the noteworthiness of considering the impact of outliers on the findings. To be more specific, if overlooked, a violation of any of the OLS assumptions would lead to a wrong estimation of standard errors. In such cases, the BOLS analysis would yield more accurate results than the OLS method because it does not rely on the underlying distributional assumptions. In the current study, a violation of no autocorrelation assumption was detected (see Table 2); this could have resulted in the erroneous rejection of the null hypothesis for the variable *“Stereotypes”*. Another methodological aspect that tends to be overlooked in applied linguistics research is the presence of outliers, which could affect the estimation of slope coefficient [49]. In the current study, three outliers were identified in the sample dataset on the Japanese language learners (see Fig 1). In the presence of outliers the QR would be a more robust method compared to the OLS because the former is based on a quantile value while the latter is based on the mean value. Combining all these methodological considerations, the BQR analysis could be a preferable method when implementing small sample research studies because the BQR analysis incorporates methodological advantages of the bootstrap and QR.

**Fig 2:**
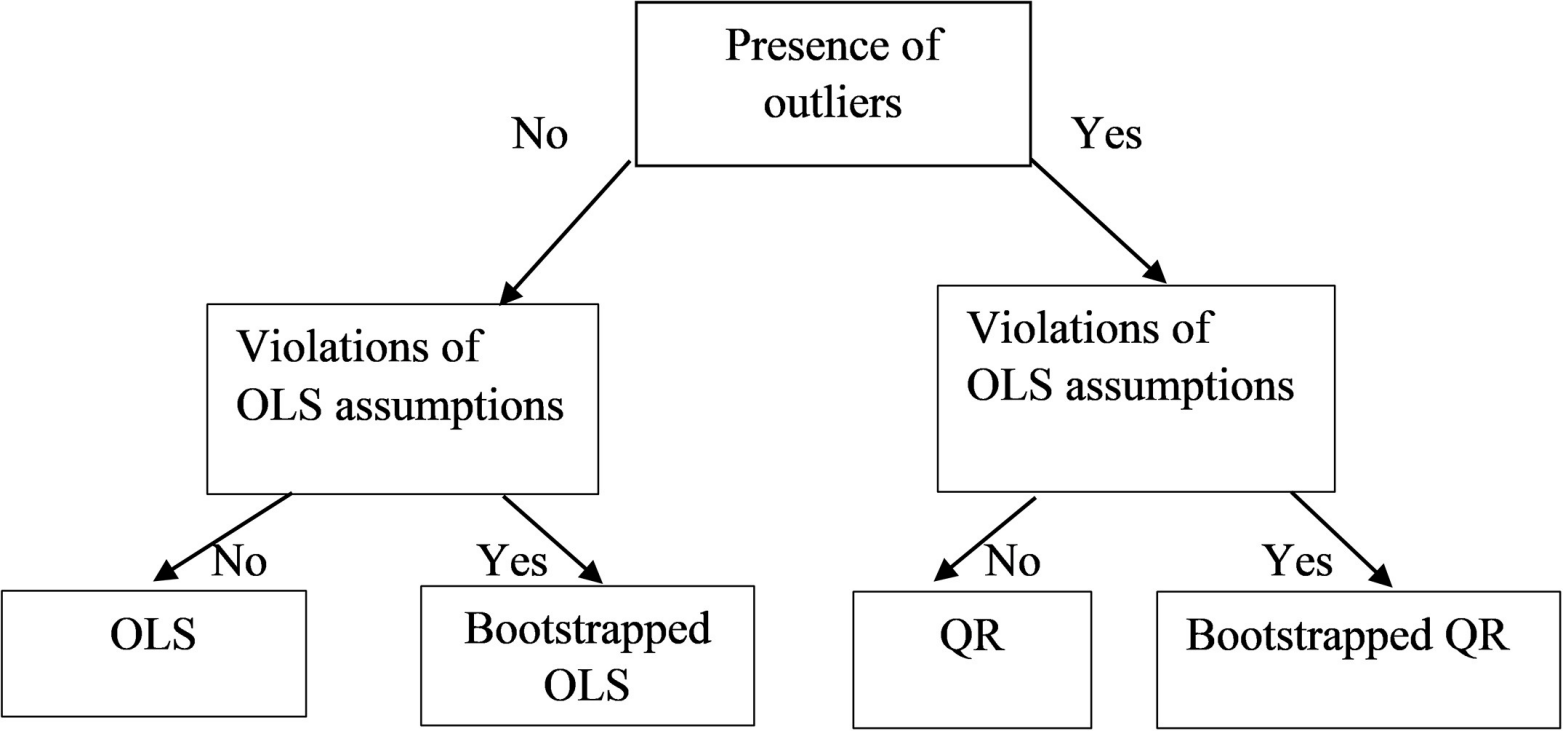
Selection of appropriate method.

In Fig 2, we propose a set of steps to assist researchers when selecting an appropriate method to analyse their small sample data. The presence of outliers and violations of OLS assumptions need to be checked in the first two steps. If there are no outliers and no violations of the assumptions, the OLS would be an appropriate method of analysis. However, if anyone of the OLS assumptions is violated then the bootstrapped OLS method could be considered. A caveat here is that the bootstrapped OLS could have a greater proportion of outliers compared to the original dataset [52]. Alternatively, if the dataset has outliers but no OLS assumptions are violated, the QR method would be appropriate. If both outliers and violations of the assumption are in evidence, the researchers might want to choose the BQR analysis.

The present study has several methodological and theoretical implications. A wider methodological implication is that employing the BQR method helps to avoid committing Type I error (i.e., a wrong rejection of the null hypothesis). As Plonsky et al. [7] demonstrated, applied linguistics research that relies on traditional parametric statistics tends to have a high rate of studies with Type I error, which compromises validity and replicability of findings reported by researchers. In other words, what the new analytical approach offers beyond the existing methods is that it enables researchers to obtain more accurate results.

As to this study’s theoretical implications, it has introduced the “*known–unknown*” continuum used in philosophy to position the statistical discussion on the (missing) link between the known sample and unknown population. Specifically for research on L2 motivation, a noteworthy implication has the finding that only the variable “*Country*” was a stable predictor at the 10 percent level of statistical significance of the language learners’ integrative orientation. As mentioned in the “Estimation Procedure” section, the significance level in the current study was set to 10 percent following a methodologists’ suggestion that this significance level be chosen for quantitative social sciences research [3, 6, 46–48].

It should be noted that the variable “*Country*” could be described as the least exacting among the study’s variables. This is because the students’ attitudes toward the TL country were measured by a thermometer-type scale that did not aim at assessing the attitudes toward any particular object, phenomenon or entity in Japan. Rather, the scale measured the students’ very general feelings toward Japan as a country. In contrast, the other two variables—“*People*” and “*Stereotypes*”—assessed more concrete attitudes, such as the attitudes toward Japanese people and the attitudes embedded in the students’ personally held and clearly discernible stereotypes about Japan. In other words, the least exacting of the three variables remained the most constant predictor of the integrative orientation. This finding supports the proposition that the integrative orientation remains one of the most difficult psychological constructs to capture [20, 21]. Overall, the findings of this study suggest that employing various set of measures and implementing appropriate statistical procedures could enable researchers to gain further insights into the nature of this elusive concept.

## Concluding remarks

In recent years, concerns have been voiced that inappropriate applications of traditional statistical methods might have unwittingly contributed to filling the literature with non-replicable findings [1, 7]. As a means to counteract the impending replication crisis, researchers continuously strive to develop methodologically advanced statistical tools [2, 6, 7]. The current study has demonstrated a practical application of the BQR analysis [6] and explained statistical reasoning behind the method. Specifically, it showed how two main problems inherent in small sample research, namely, the violation of distributional assumptions and the presence of outliers, could be mitigated when using the BQR method.

Along the “*known—unknown*” continuum discussed in the introductory section, the BQR analysis could be identified as the “*unknown known*” type. In this method, the known empirically generated sampling distribution is used to construct the confidence intervals (CIs) for the unknown population parameters. Therefore, estimations in the BQR method do not have to rely on the underlying distributional assumptions. Besides of this methodological advantage, the BQR procedure is based on the quantile regression (QR) estimation method which is more robust against the presence of outliers. It is much hoped that the BQR method would help to achieve valid and replicable results and that this method could be adopted in future small sample studies in applied linguistics and other human sciences.

## Supporting information

S1 Table Data used for the analysis. (TXT)

S1 Code R code for OLS analysis. (TXT)

S2 Code R code for bootstrapped OLS. (TXT)

S3 Code R code for QR. (TXT)

S4 Code R code for bootstrapped QR. (TXT)

## References

[1] Skidmore ST, Thompson B. Statistical techniques used in published articles: A historical review of reviews. Educational and Psychological Measurement. 2010; 70: 777–795. 10.1177/0013164410379320

[2] Matthews RAJ. Beyond ‘significance’: Principles and practice of the Analysis of Credibility. Royal Society Open Science. 2018; 5: 171047 10.1098/rsos.171047 29410818 PMC5792895

[3] Larson-Hall J. A guide to doing statistics in second language research using SPSS and R. New York: Routledge; 2016.

[4] Larson-Hall J, Herrington R. Improving data analysis in second language acquisition by utilizing modern developments in applied statistics. Applied Linguistics. 2010; 31(3): 368–390. 10.1093/applin/amp038

[5] Larson-Hall J. Our statistical intuitions may be misleading us: Why we need robust statistics. Language Teaching. 2012; 45(4): 460–474. 10.1017/S0261444811000127

[6] Nikitina L, Furuoka F. Expanding the methodological arsenal of applied linguistics with a robust statistical procedure. Applied Linguistics. 2018; 39(3): 422–428. 10.1093/applin/amx026

[7] Plonsky L, Egbert J, Laflair GT. Bootstrapping in applied linguistics: Assessing its potential using shared data. Applied Linguistics. 2015; 36(5): 591–610. 10.1093/applin/amu001

[8] Žižek S. Event. London, UK: Penguin Books; 2014.

[9] Amemiya T. Introduction to statistics and econometrics. London, UK: Harvard University Press; 1994.

[10] Keuzenkamp HA. Probability, econometrics and truth. Cambridge, UK: Cambridge University Press; 2000.

[11] Gorjian B, Hayati A, Pourkhoni P. Using Praat software in teaching prosodic features to EFL learners. Procedia–Social and Behavioral Sciences. 2013; 84: 34–40. 10.1016/j.sbspro.2013.06.505

[12] Brown JD. Understanding research in second language learning: A teacher’s guide to statistics and research design. Cambridge, UK: Cambridge University Press; 1988.

[13] Goldstein H, Lynn P, Muniz-Terrera G, Hardy R, O’Muircheartaigh C, Skinner C, et al Population sampling in longitudinal surveys. Longitudinal and Life Course Studies. 2015; 6(4): 447–475. 10.14301/llcs.v6i4.345

[14] Bai H, Sivo SA, Pan W, Fan X. Application of a new resampling method to SEM: A comparison of S-SMART with the bootstrap. International Journal of Research & Method in Education. 2016; 39(2): 194–207. 10.1080/1743727X.2015.1056135

[15] Chernozhukov V, Hansen C, Jansson M. Finite sample inference for quantile regression models. Journal of Econometrics. 2009; 152: 93–103. 10.1016/j.jeconom.2009.01.004

[16] Chen F, Chalhoub-Deville M. Principles of quantile regression and an application. Language Testing. 2014; 31(1): 63–87. 10.1177/0265532213493623

[17] Gardner RC, Lambert WE. Motivational variables in second-language acquisition. Canadian Journal of Experimental Psychology. 1959; 13(4): 266–272. 10.1037/h0083787 13855818

[18] Gardner RC, Lambert WE. Attitudes and motivation in second-language learning. Rowley, MA: Newbury House Publishers; 1972.

[19] Gardner RC. Social psychology and second language learning: The role of attitudes and motivation. London, UK: Edward Arnold; 1985.

[20] Nikitina L, Zuraidah MD, Loh SC. Construction and validation of a questionnaire on language learning motivation. Zbornik Instituta za Pedagoska Istrazivanja. 2016; 48(2): 284–300. 10.2298/ZIPI1602284N

[21] Papi M. The L2 motivational self system, L2 anxiety, and motivated behavior: A structural equation modeling approach. System. 2010; 38(3): 467–479. 10.1016/j.system.2010.06.011

[22] Spencer-Rodgers J. Consensual and individual stereotypic beliefs about international students among American host nationals. International Journal of Intercultural Relations. 2001; 25(6): 639–657. 10.1016/S0147-1767(01)00029-3

[23] Gallagher HC. Willingness to communicate and cross-cultural adaptation: L2 communication and acculturative stress as transaction. Applied Linguistics. 2013; 34(1): 53–73.

[24] Hessel G. From vision to action: Inquiring into the conditions for the motivational capacity of ideal second language selves. System. 2015; 52: 103–114.

[25] Gu MM, Cheung DSP. Ideal L2 self, acculturation, and Chinese language learning among South Asian students in Hong Kong: A structural equation modelling analysis. System. 2016; 57: 14–24.

[26] Zhang D. Derivational morphology in reading comprehension of Chinese-speaking Learners of English: A longitudinal structural equation modeling study. Applied Linguistics. 2017; 38(6): 871–895. 10.1093/applin/amv072

[27] Davidson R, MacKinnon JG. Econometric theory and methods. Oxford: Oxford University Press; 2004.

[28] Hayashi F. Econometrics. Princeton, USA: Princeton University Press; 2000.

[29] Harvey AC. The econometric analysis of time series. Oxford, UK: Philip Allen; 1981.

[30] Gujarati DN, Porter DC. Basic econometrics. Singapore: McGraw Hill; 2009.

[31] Stuckler D, Basu S, Suhrcke M, Coutts A, McKee M. The public health effect of economic crises and alternative policy responses in Europe: An empirical analysis. Lancet. 2009; 374(9686): 315–323. 10.1016/S0140-6736(09)61124-7 19589588

[32] Efron B. Bootstrap methods: Another look at the Jackknife. Annals of Statistics. 1979; 7(1): 1–26. 10.1214/aos/1176344552

[33] Efron B. Nonparametric estimates of standard error: The Jackknife, the bootstrap and other methods. Biometrika. 1981; 68(3): 589–599. 10.1093/biomet/68.3.589

[34] Efron B, Tibshirani R. Bootstrap methods for standard errors, confidence intervals, and other measures of statistical accuracy. Statistical Science. 1986; 1(1): 54–75. 10.1214/ss/1177013815

[35] Guan W. From the help desk: Bootstrapped standard errors. Stata Journal. 2003; 3(1): 71–80.

[36] Maddala GS, Kim IM. Unit roots, cointegration and structural change. Melbourne: Cambridge University Press; 1998.

[37] Poi BP. From the help desk: Some bootstrapping techniques. Stata Journal. 2004; 4(3): 312–328.

[38] Hall P, Wilson SW. Two guidelines for bootstrap hypothesis testing. Biometrics. 1991; 47(2): 757–762. 10.2307/2532163

[39] Koenker R, Bassett G. Regression quantiles. Econometrica. 1978; 46(1): 33–50. 10.2307/1913643

[40] Jarque CM, Bera AK. Efficient tests for normality, homoscedasticity and serial independence of regression residuals. Economics Letters. 1980; 6(3): 255–259. 10.1016/0165-1765(80)90024-5

[41] Breusch TS, Pagan AR. A simple test for heteroscedasticity and random coefficient variation. Econometrica. 1979; 48: 1287–1294. 10.2307/1911963

[42] Godfrey LG. Testing for multiplicative heteroscedasticity. Journal of Econometrics. 1978a; 8: 227–236. 10.1016/0304-4076(78)90031-3

[43] Godfrey LG. Testing against general autoregressive and moving average error models when the regressors include lagged dependent variables. Econometrica. 1978b; 46: 1293–1301. 10.2307/1913829

[44] Breusch TS. Testing for autocorrelation in dynamic linear models. Australian Economic Papers. 1978; 17: 334–355. 10.1111/j.1467-8454.1978.tb00635.x

[45] Mizumoto A, Plonsky L. R as a Lingua Franca: Advantages of using R for quantitative research in applied linguistics. Applied Linguistics. 2016; 37(2): 284–29. 10.1093/applin/amv025

[46] Kline R. Beyond significance testing: reforming data analysis methods in behavioral research. Washington, DC: American Psychological Association; 2004.

[47] Murphy KR, Myors B. Statistical power analysis. Mahwah, NJ: Erlbaum; 2004.

[48] Rasinger SM. Quantitative research in linguistics: an introduction. London: Bloomsbury Academic; 2013.

[49] Gujarati DN, Porter DC. Basic econometrics. Singapore: McGraw Hill; 2009.

[50] Huber PJ. Robust statistics. New York: John Wiley & Sons; 2004.

[51] Cohen JE. Statistical power analysis for the behavioural sciences. Hillsdale, NJ: Lawrence Erlbaum Associates; 1988.

[52] Salibian-Barrera M, Zamar RH. Bootstrapping robust estimates of regression. Annals of Statistics. 2002; 30: 556–82.

